# Anti-filarial Activity of Antibiotic Therapy Is Due to Extensive Apoptosis after *Wolbachia* Depletion from Filarial Nematodes

**DOI:** 10.1371/journal.ppat.1002351

**Published:** 2011-11-03

**Authors:** Frederic Landmann, Denis Voronin, William Sullivan, Mark J. Taylor

**Affiliations:** 1 Department of Molecular, Cell and Developmental Biology, Sinsheimer Labs, University of California, Santa Cruz, California, United States of America; 2 Molecular and Biochemical Parasitology, Liverpool School of Tropical Medicine, Pembroke Place, Liverpool, United Kingdom; Stanford University, United States of America

## Abstract

Filarial nematodes maintain a mutualistic relationship with the endosymbiont *Wolbachia*. Depletion of *Wolbachia* produces profound defects in nematode development, fertility and viability and thus has great promise as a novel approach for treating filarial diseases. However, little is known concerning the basis for this mutualistic relationship. Here we demonstrate using whole mount confocal microscopy that an immediate response to *Wolbachia* depletion is extensive apoptosis in the adult germline, and in the somatic cells of the embryos, microfilariae and fourth-stage larvae (L4). Surprisingly, apoptosis occurs in the majority of embryonic cells that had not been infected prior to antibiotic treatment. In addition, no apoptosis occurs in the hypodermal chords, which are populated with large numbers of *Wolbachia*, although disruption of the hypodermal cytoskeleton occurs following their depletion. Thus, the induction of apoptosis upon *Wolbachia* depletion is non-cell autonomous and suggests the involvement of factors originating from *Wolbachia* in the hypodermal chords. The pattern of apoptosis correlates closely with the nematode tissues and processes initially perturbed following depletion of *Wolbachia*, embryogenesis and long-term sterilization, which are sustained for several months until the premature death of the adult worms. Our observations provide a cellular mechanism to account for the sustained reductions in microfilarial loads and interruption of transmission that occurs prior to macrofilaricidal activity following antibiotic therapy of filarial nematodes.

## Author Summary

Filarial nematodes are a group of pathogenic helminth parasites that include those responsible for the diseases, lymphatic filariasis and onchocerciasis. These nematodes have evolved a mutualistic association with the intracellular bacterial symbiont, *Wolbachia*, providing a novel approach to their treatment and control using antibiotics. Why depletion of *Wolbachia* leads to anti-filarial activity remains unknown. Here we show that depletion of the bacteria leads to a rapid and sustained induction of cell death through apoptosis of reproductive cells, developing embryos, microfilariae and developing larvae. Apoptosis surprisingly affects embryonic cells other than those infected with the bacteria, leading us to conclude that the *Wolbachia* populations in the adult hypodermis, rather than the few bacteria present in the developing embryos, are essential to avoid apoptosis. Our observation provides a mode of action to account for some of the key anti-filarial activities of antibiotic treatment of filarial nematodes and identifies one of the biological processes that form the basis of this mutualistic symbiosis.

## Introduction

The majority of filarial nematodes host *Wolbachia* bacteria in a mutualistic symbiotic association. In adult worms, the endosymbionts are situated in the hypodermal lateral chord cells, located within host-derived vacuoles. In females, *Wolbachia* are also found in the ovaries, oocytes and developing embryos within the uteri [1]–[4].

The mutualistic association of *Wolbachia* in filarial nematodes has been exploited as a novel approach to the treatment of lymphatic filariasis caused by *Wuchereria bancrofti* and *Brugia malayi* and onchocerciasis caused by *Onchocerca volvulus* [5]. The use of tetracyclines or rifamycins to deplete *Wolbachia* leads to an arrested development of larval and embryonic stages resulting in permanent sterilization of adult female worms [6]. The adult parasites die prematurely after 1–2 years following depletion of *Wolbachia,* compared to their typical lifespan of 10–14 years, delivering for the first time a safe and potent macrofilaricidal treatment for filariasis [5].

Although the effects of *Wolbachia* depletion on the development, fertility and viability of filarial nematodes has been documented (reviewed in [7]), the reason why depletion of *Wolbachia* leads to these anti-filarial outcomes is unknown. Here we have used whole mount confocal microscopy to observe the consequences of *Wolbachia* depletion on host nematode cellular and nuclear structure. Our observations reveal an extensive and profound development of apoptosis in germline cells and embryos following antibiotic depletion of *Wolbachia*, which occurs soon after bacterial depletion in *B. malayi* and is sustained for at least 21 months in *O. volvulus.* We find extensive apoptosis even in cells that had not been infected with *Wolbachia* prior to antibiotic treatment. Nuclear structure in most somatic tissues remains intact, although disruption of cytoskeleton arrangement occurs in the lateral chord cells, where the vast majority of the bacteria reside.

## Results

### Morphological alteration of *in vivo* treated *Brugia malayi* adult worms

**Figure 1:**
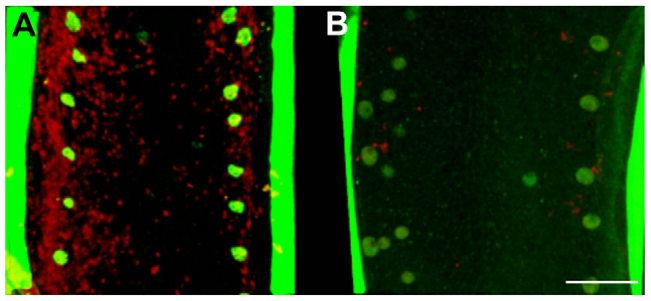
*In vivo* tetracycline treatment dramatically reduces the *Wolbachia* population in adult *B. malayi* females. Female *B. malayi* lateral chords from un-treated (A) or tetracycline-treated (B) jirds. Total DNA is revealed with propidium iodide (red), host nuclei are counterstained with an anti acetylated histone H4 (green), therefore the red foci reveal only *Wolbachia.* The chords are flanked by muscle quadrants stained with phalloidin (green). Scale bar  = 100 µm.

To investigate the contribution of *Wolbachia* to its filarial host fitness and fertility, jirds infected with *B. malayi* were treated with tetracycline (2.5 mg/ml in drinking water), for a period of 6 weeks. Parasites were recovered from the peritoneal cavity at 8 weeks post-treatment. Female worms from treated and non-treated jirds were collected and stained for the presence of *Wolbachia* in the lateral chords, the somatic tissue that they populate in the adult. As expected a dramatic reduction of the bacterial population was observed in this tissue after the antibiotic treatment (Figure 1A, B). *Wolbachia* depletion was confirmed and quantified by quantitative PCR using a ratio of single copy genes: *wsp* (for *Wolbachia*) and *gst* (for *B. malayi*) [8], which showed an 99% reduction of bacterial load in treated adult female worms and in 14 day old L4 larvae.

**Figure 2:**
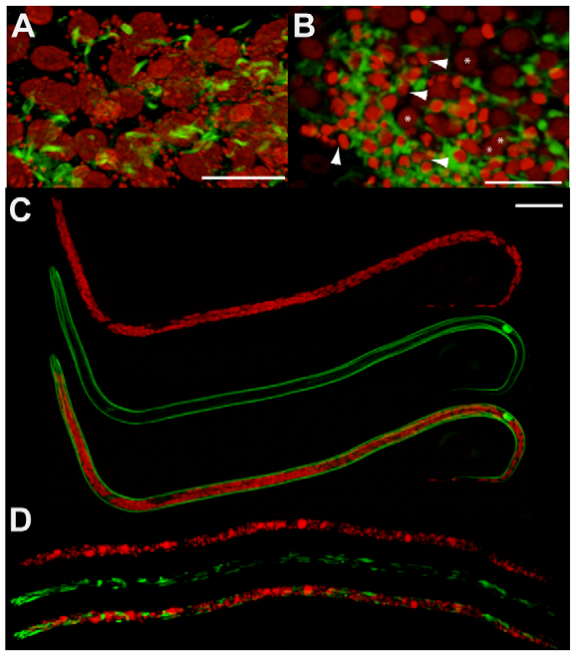
Pyknotic nuclei and morphology defects observed in the germline and intrauterine microfilariae. Germline cells-containing ovaries (A, B) and microfilariae obtained from uteri (C, D) were stained for DNA (propidium iodide, red) and actin (phalloidin, green). Worms dissected from un-treated animals (A, C), and from animals treated with tetracycline (B, D). In (A), propidium iodide reveals germline nuclei surrounded by *Wolbachia* (appearing as small red foci), while *Wolbachia* are absent in (B). In (B), arrowheads point towards some pyknotic nuclei, with a condensed chromatin appearing brighter with propidium iodide stain, while stars indicate germ cell nuclei with a normal morphology. Note that depending on the area observed in the ovary, wild type nuclei can slightly vary in volume, as shown in images (A) and (B). Scale bar  = 20 µm.

Morphological defects in somatic tissues and in the germline, based on DNA and actin staining were investigated using confocal microscopy on whole mount nematodes. Numerous pyknotic nuclei were observed throughout the ovaries and uteri in the female germline through to the later stages of embryogenesis and ‘stretched’ microfilariae in treated worms (Figure 2). For example, condensed and fragmented oogoniae nuclei surrounded by an intense actin staining were observed in the treated females, suggesting a reduction of the cytoplasmic volume (Figure 2 A, B). Most of the intrauterine ‘stretched’ microfilariae that resulted from a completed embryogenesis showed morphological defects such as abnormal muscle quadrants, associated with pyknotic nuclei (Figure 2 C, D).

**Figure 3:**
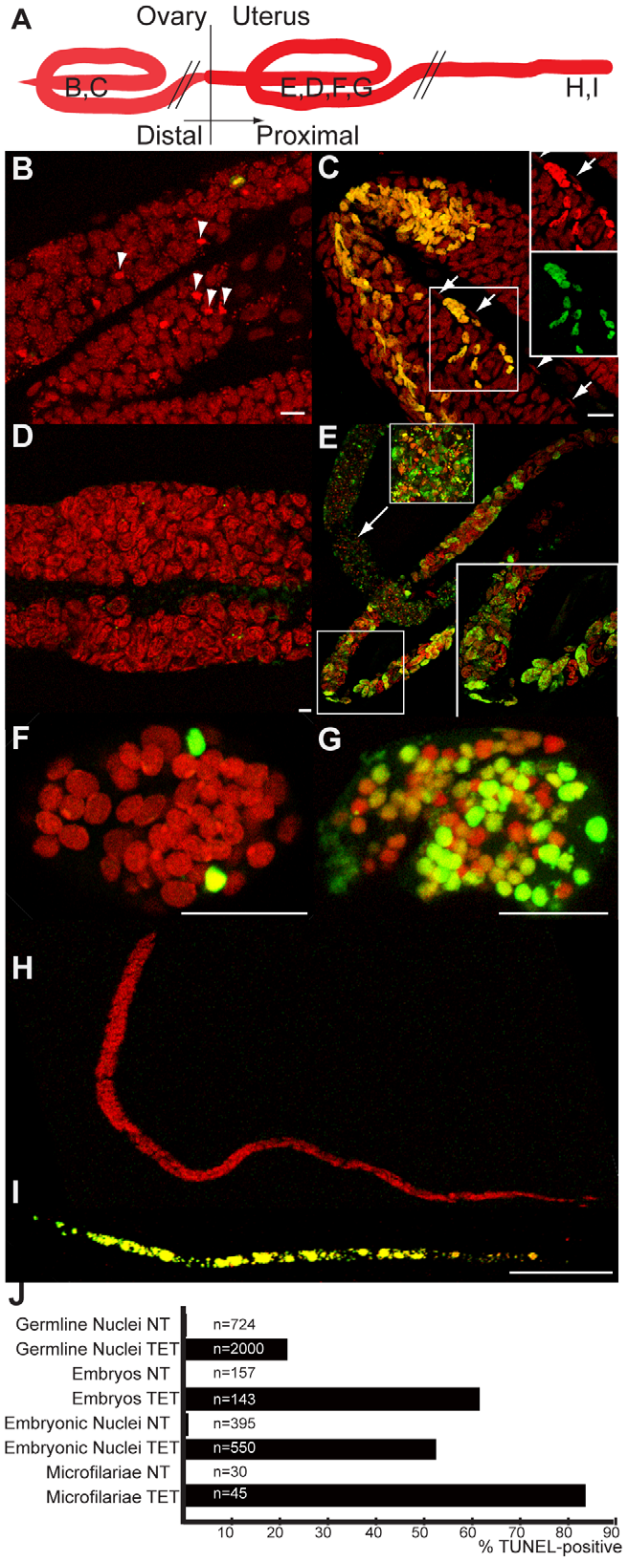
*In vivo* tetracycline treatment leads to apoptosis in adult worm reproductive tissues. (A) Schematic drawing of one female reproductive tract, representing the approximate localization of the different sections observed in the TUNEL assay. TUNEL experiments showing the DNA (PI in red) and incorporated fluorescein-dUTP (green), in samples from non-treated (B, D, F, H) or tetracycline-treated (C, E, G, I) *B. malayi* females. Mitotic proliferation zone in the distal ovary (B, C). Arrowheads indicate mitotic nuclei, arrows point to somatic gonad nuclei. (D) Uterus filled with elongating embryos. (E) Fertilization area in the distal uterus in the top left corner, and proximal uterus filled (in diagonal) with developing embryos. (F, G) Single developing embryos. (H, I) Intrauterine microfilariae extracted from proximal uteri. (J) TUNEL quantification. For each un-treated (NT) or tetracycline-treated (TET) sample, TUNEL-positive nuclei or embryos were counted and expressed as a percentage of total nuclei or embryos (based on DNA staining with PI). For the embryonic count (“embryos NT”, “embryos TET”), embryos with a number of TUNEL positive nuclei equal or less than 2 positive nuclei were considered as negative embryos. All the intrauterine microfilariae found TUNEL-positive in the TET sample had every nuclei TUNEL-positive (I). Scale bar  = 15 µm.

Since pyknosis is a hallmark of cell death, the next step was to determine whether the depletion of *Wolbachia* induced apoptosis as detected using the TUNEL assay [9]. TUNEL allows detection of apoptosis-caused DNA fragmentation by incorporation of fluorescent dUTP to DNA 3’ OH free ends. The germline in ovaries and the embryos in the uterus were examined and the proportion of each stage undergoing apoptosis was quantified (Figure 3 A to J). In non-treated females, apoptosis at the level of germline nuclei is a rare event (0.4%, n = 724), while apoptotic nuclei were widely detected as patches in the ovaries of treated females (22%, n = 2000 nuclei from a total of 4 treated females) (Figure 3 B, C, J). Apoptotic nuclei became more numerous as the uteri filled with embryos in treated females, while no abnormal apoptosis was detected in the control non-treated females (Figure 3 D, E and J).

In embryos, a few cells were TUNEL positive in the untreated control samples (Figure 3 F, J). This apoptosis is likely to be developmentally programmed. In *Caenorhabditis elegans*, where it is well characterized, about 12% of the total adult somatic cells undergo programmed cell death during development [10]. In contrast, the majority of the blastomeres of TUNEL-positive embryos from treated females were apoptotic (Figure 3 G, J, 53%, n = 550).

We finally observed the intrauterine ‘stretched’ microfilariae extracted from the proximal uteri. No apoptosis was detected in the control samples, whereas most of the ‘stretched’ microfilariae from treated females were entirely undergoing apoptosis (Figure 3 H, I, J 83%, n = 45). Apoptosis appeared to be cumulative, with more and more embryos affected as development progresses.

**Figure 4:**
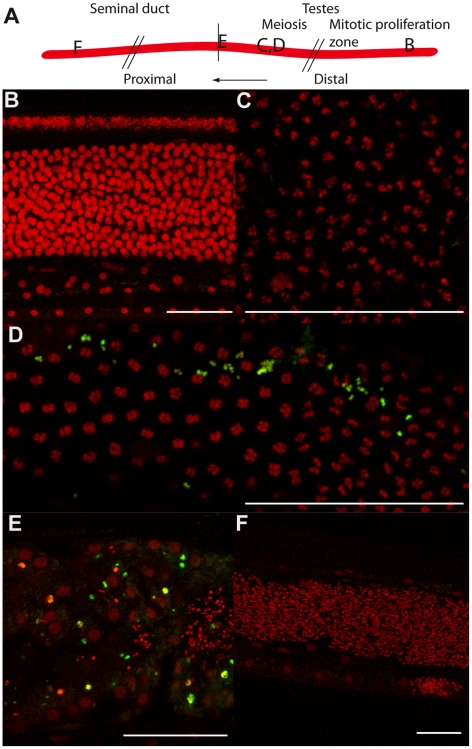
Tetracycline treatment induces detectable apoptosis during spermatogenesis. TUNEL experiments showing the DNA (PI in red) and incorporated fluorescein-dUTP (green), in samples from un-treated (B, C) or tetracycline-treated (D to F) *B. malayi* males. (A) Schematic drawing of the male reproductive tract, representing the approximate localization of the different sections observed in the TUNEL assay. (B) Mitotic proliferation zone of spermatogoniae. (C, D) Synaptonemal complexes in meiosis I. (E) Proximal testes. (F) Seminal duct filled with mature spermatocytes. Scale bar  = 100 µm.

To better understand the contribution of the *Wolbachia* in the lateral chord versus the few *Wolbachia* present in oocytes, and subsequently present in the embryo by maternal transmission (from early to mid-embryogenesis we found an average of 70 *Wolbachia* +/−12 (n = 10) per embryo), we examined *B. malayi* males, which are devoid of *Wolbachia* in the germline. We performed a TUNEL assay in males obtained from the same jirds. While no apoptosis was detected in control males, we observed a small number of apoptotic events in irregular patches in the germline of treated males, suggesting that the *Wolbachia* in the chords play a role in preventing apoptosis in the male germline (Figure 4 A to F).

In females, the chords are larger than in males and contain ∼10 fold more *Wolbachia* in worms 6 months or older, although they begin their adult lives with equivalent numbers and ratios [11]. The chords are closely apposed to the uteri, and this adjacency possibly facilitates the supply of nutrients or critical metabolites to the growing embryos [12]. The *Wolbachia* present in the female lateral chords may therefore have a more important role than in males, and their contribution may be crucial to avoid apoptosis during female germline and embryonic development.

**Figure 5:**
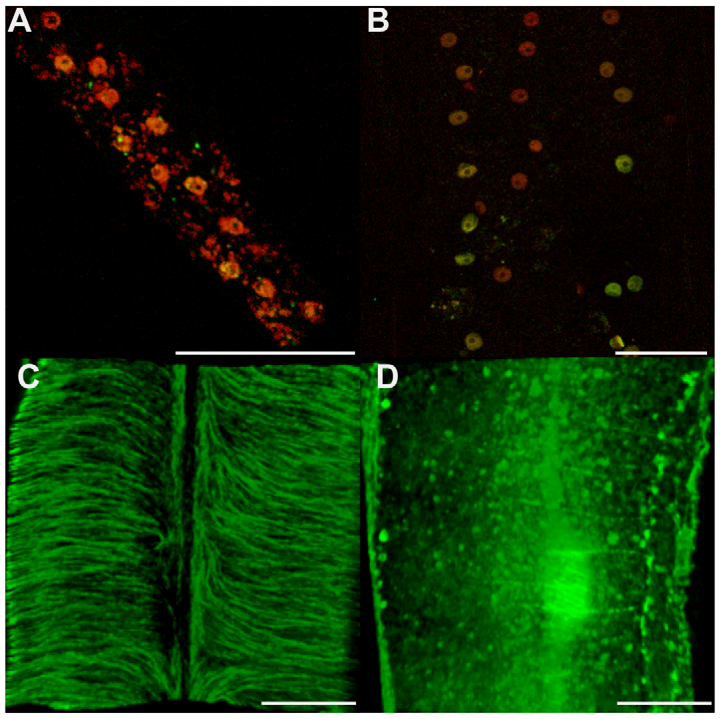
Cytoskeleton defects are revealed in somatic tissues, without apoptotic phenotypes. Female *B. malayi* lateral chords from un-treated (A, C) or tetracycline-treated (B, D) jirds. No pyknotic nuclei were detected, and the TUNEL levels were similar in both samples (A, B). (C, D) Apical microtubule network. Scale bar  = 100 µm.

The effect of *Wolbachia* depletion on the nuclei and cytoskeleton morphologies in the lateral chords in treated and non-treated females was investigated next (Figures 1 and 5). Lateral chord cells are syncytial and the prominent rows of nematode nuclei are easily observed. The lateral chord nuclei showed no evidence of pyknosis or any difference in TUNEL staining in either treated or untreated worms (Figure 5 A, B). This suggests that that the loss of *Wolbachia* in somatic tissues, does not lead to apoptosis of the lateral chord cells. However, the cortical microtubule network, circumferentially oriented in loose bundles in control samples, was disrupted in treated females (Figure 5 C, D). These cytoskeleton defects may impair the chords function in supplying nutrients/metabolites to the germline and the developing embryos.

**Figure 6:**
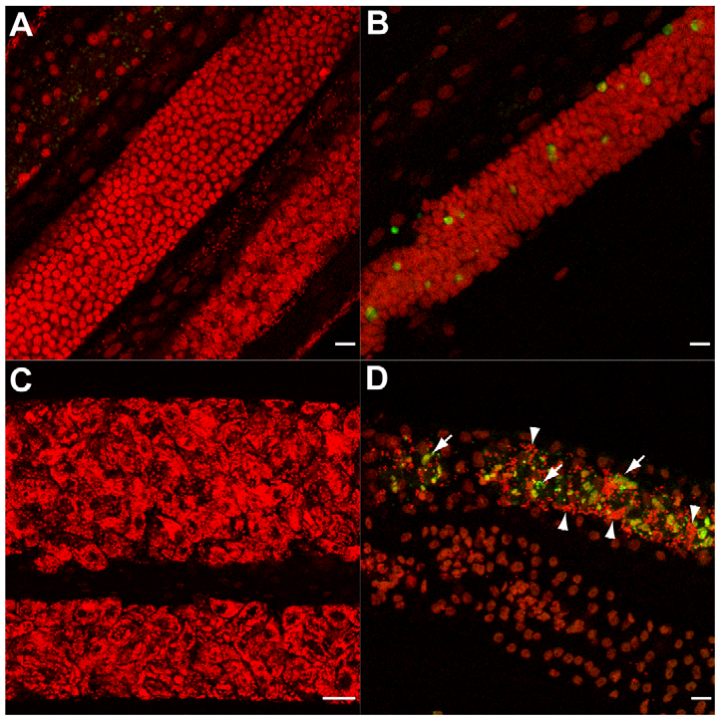
Doxycycline treatment leads to apoptosis *in vitro.* (A, C) control worms and treated worms (B, D) were TUNEL assayed (green) and stained for DNA (PI in red). (A, B) Germ cells in mitotic proliferation in the ovaries. (C) Proximal uteri filled with developing embryos. (D) Apoptotic oocytes and early embryos (arrows) in a distal uterus, surrounded by sperm cells (arrowheads). Scale bar  = 15 µm.

In order to determine whether the observed increase in apoptosis in germline cells and embryos was dependent on any mammalian host factors, we cultured untreated female and male worms *in vitro* with doxycycline (8 µM) for a period of five days [8]. Worms were TUNEL-assayed at day 1, 2, 4 and 5 post-treatment. No differences were seen between control and treated worms until day 4. On days 4 and 5, doxycycline-treated worms showed abnormal apoptosis in germ cells, during fertilization, and in young embryos (Figure 6). Based on this *in vitro* doxycycline treatment, we concluded that the *in vivo* tetracycline-induced *Wolbachia* depletion could cause apoptosis independently of any mammalian host derived factors.

### Apoptosis in *Brugia malayi* microfilaria following tetracycline treatment

Following intra-peritoneal infection of jirds with *B. malayi,* the adults mate and release microfilariae, which remain confined to the peritoneal cavity. Because microfilariae accumulate in the peritoneal cavity, a proportion of those recovered will include moribund or dead parasites. Therefore, we first determined the basal level of apoptosis using the TUNEL assay in microfilaria from untreated jirds using the following criteria: high (more than 20 nuclei), medium (5–20 nuclei) and low (less than 5 nuclei) levels of apoptotic-positive nuclei per single microfilariae. One hundred microfilariae from each treatment group were analyzed. Eighty three percent of microfilaria from untreated control groups had none or less than 5 apoptotic positive nuclei, 14% had a medium number of apoptotic positive nuclei (5–20) and 3% contained the highest level (>20). In the tetracycline treated group there was a 2.6 fold increase in the proportion of microfilaria with a medium number of apoptotic nuclei (34%) and a 7.3 fold increase in the proportion of microfilariae with the highest level of apoptotic nuclei (22%) (Table 1). So, although we observed an increase in the levels of apoptosis in released microfilariae, a significant proportion (44%) only show minimal or no induction of apoptosis, unlike intrauterine ‘stretched’ microfilariae where the evidence of apoptosis is extensive and widespread affecting the vast majority of the terminal development stage in the uterus.

**Table 1:** Percentage of *B. malayi* microfilariae showing different levels of apoptotic positive nuclei.

	Percentage of apoptotic positive nuclei per microfilaria		
	<5	5–20	>20
Control untreated *B. malayi*	83.4%	13.5%	3.1%
Tetracycline treated *B. malayi*	43.8%	34.2%	22.0%

### Increases in cell death protein-3 (*ced-3*) gene expression and levels of activated CED-3 protein are observed in *Wolbachia-*depleted parasites

**Figure 7:**
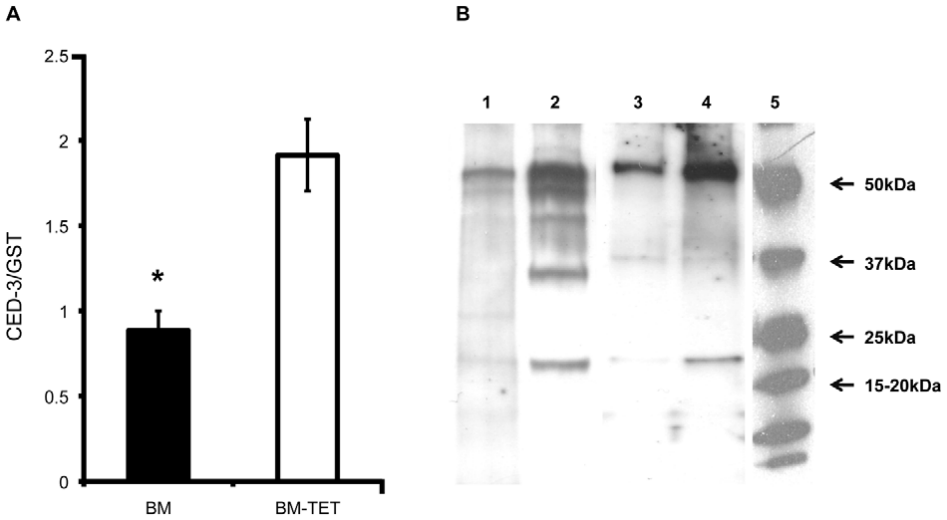
Increased expression of *ced-*3 gene and activation of CED-3 protein in tetracycline treated *B. malayi*. A) *Ced*-3 gene expression level normalized by expression level of *gst* in BM (*B. malayi*) and BM-TET (tetracycline treated *B. malayi*) adult females. B) Western blot detection of CED-3 in microfilaria and 14 day old L4 larvae. Lanes 1-2, microfilariae from untreated control (1) and tetracycline treated (2). Lanes 3-4 lanes, L4 larvae, untreated control (3) and tetracycline treated (4). Activated (cleaved) CED-3 is more abundant in microfilariae and L4 larvae from treated jirds compared to untreated controls. Lane 5, molecular weight markers.

To further investigate the induction of apoptosis following depletion of *Wolbachia,* we analyzed gene expression and protein profiles of cell death protein-3 (*ced*-3), a homologue of human Caspase-3. The relative level of cell death protein-3 (*ced*-3) gene expression was significantly increased in tetracycline treated females (p<0.01, n = 6 worms) compared with untreated controls (Figure 7 A). We performed a western blot analysis of CED-3 protein extracted from microfilariae and 14 day old L4 larvae obtained *ex vivo*. Caspase-3 was detected in the inactive form (∼50 kDa) and as cleaved activated forms (from 47 to 19 kDa). Figure 7B shows an increased amount of inactive and cleaved CED-3 forms in the tetracycline treated samples compared with controls, demonstrating the activation of CED-3 and it’s over expression in microfilaria and L4 larvae following depletion of *Wolbachia.*

### Apoptosis is observed by TUNEL in *Onchocerca volvulus* from nodule biopsies of doxycycline-treated human patients

One of the outcomes of doxycycline therapy of filarial parasites is the long-term sterilization through blockage of embryogenesis leading to sustained reductions in microfilarial loads post treatment. In order to investigate whether this long-term sterility was a direct result of sustained embryonic apoptosis, we analyzed adult *O. volvulus* obtained from a field trial of doxycycline in Cameroon [13].

**Figure 8:**
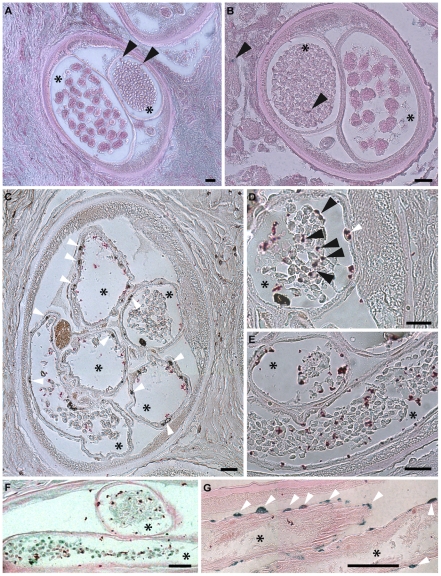
Apoptosis and apoptotic bodies are detected in *O. volvulus* tissues from human nodules of doxycycline treated patients. A, B. Cross-sections of adult female worm showing absence of apoptosis and intact embryonic inter-uterine stages (oocytes, pretzel stages, coiled embryonic microfilariae). C-G. Cross-sections of adult female worms depleted of *Wolbachia* showing extensive apoptosis of germline and early embryonic cells and uterine epithelial cells. Stars label inter-uterine content, black arrowheads label apoptotic germline and early embryonic cells as well as human cells surrounding the worm, white arrowheads point to somatic cells, such as epithelial cells surrounded uteri. Scale bar  = 20 µm.

Paraffin sections of *O. volvulus* nodules collected from 6 different patients of each treatment group (doxycycline treated and placebo treated, see Materials and Methods) were investigated using the TUNEL assay. In all samples obtained from the doxycycline treated group, numerous apoptotic-positive cells were observed in germline or early embryonic cells and in somatic nuclei of the surrounding uterus within adult female parasites (Figure 8). Other uterine embryonic stages were absent due to the blockage of embryogenesis observed following doxycycline therapy [14]. In contrast, samples collected from placebo treated patients, showed only very occasional evidence of apoptotic-cells in the different embryonic developmental stages *in utero* and no evidence of apoptosis in the uterine wall (Figure 8 A, B).

## Discussion

### *Wolbachia* depletion induces apoptosis in germline and somatic tissues

Our observations show that an extensive apoptosis of adult germline cells, embryos and somatic cells of microfilariae occurs following the depletion of *Wolbachia* from filarial nematodes. The development of apoptosis occurs soon after the depletion of bacteria with tetracycline in experimental infections of *B. malayi* in animals and *in vitro.* These observations were confirmed by evidence of activation of Cell death protein-3 in treated adult females, microfilaria and L4 larvae. Furthermore, apoptosis is observed in the germline cells and uterine tissues of *O. volvulus* at least 21 months following antibiotic treatment of people with onchocerciasis. These observations are consistent with the known anti-filarial effects of *Wolbachia* depletion on the rapid and sustained blockage of embryogenesis, the decline of microfilarial loads and the interruption of transmission to vectors and the arrested development of larvae to adults in the mammalian host [7], [14]–[16]. Previous studies showing that antibiotic treatment of the *Wolbachia-*free filarial nematode, *Acanthocheilonema viteae* has no effect on the viability or biological processes of this species [17], supports our conclusion that the observed apoptosis is due to the loss of *Wolbachia* rather than a direct effect of tetracycline treatment. The lack of apoptosis in lateral chord cells and other somatic tissues suggests the event is not a global consequence of *Wolbachia* depletion, which is consistent with the long and gradual decline in the viability of adult worms.

In the case of onchocerciasis, permanent sterilization of adult females is a therapeutically attractive outcome, as this blocks the release of the skin dwelling microfilariae, the developmental stage that gives rise to skin and eye disease [3], [7], [14]. In lymphatic filariasis, where the adult stage is responsible for disease pathogenesis of damage to the lymphatics, the removal of adult worms is required. In both cases the permanent sterilization of adult worms will deliver important benefits for the interruption of parasite transmission via blood feeding insect vectors [5], [18].

### Non cell-autonomous effects of *Wolbachia* depletion

**Figure 9:**
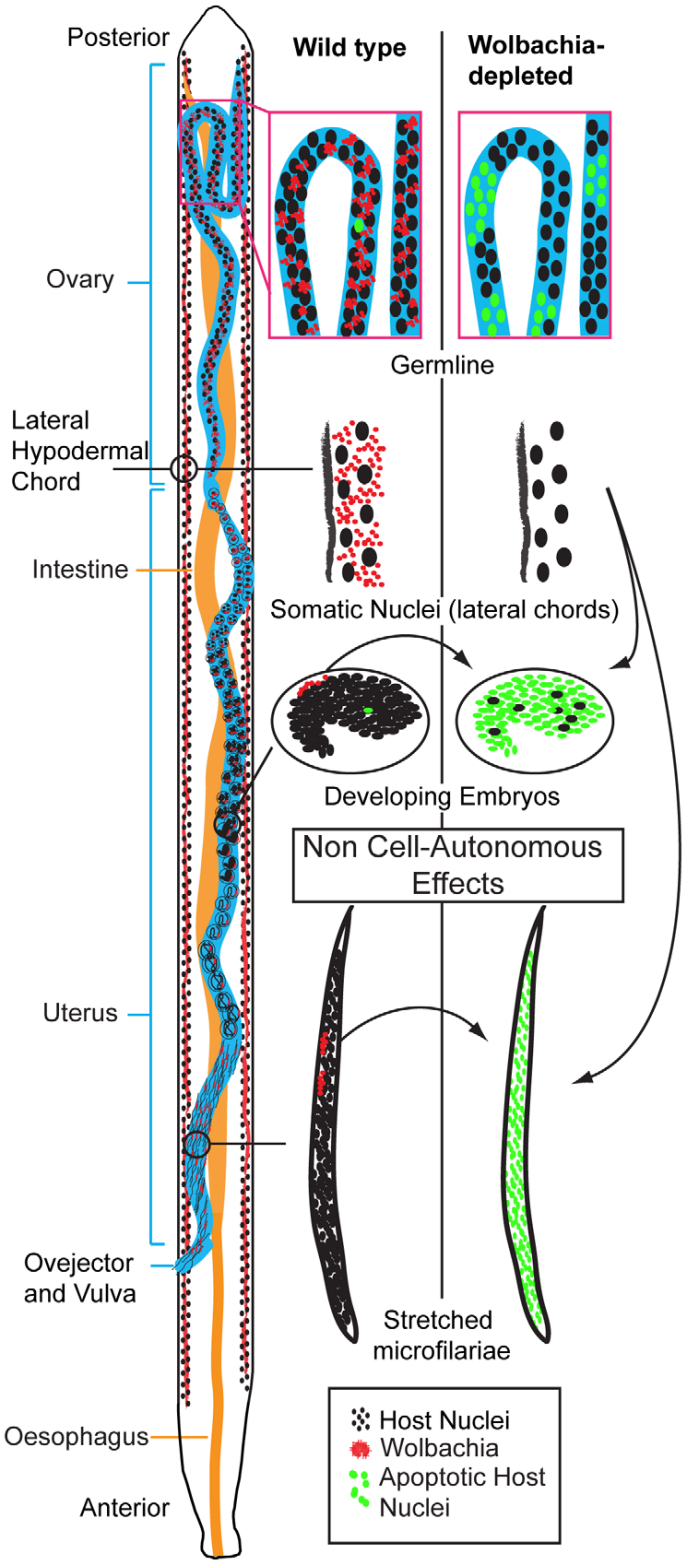
*Wolbachia*-dependent non cell-autonomous apoptosis. Schematic drawing of a *B. malayi* female focusing on the reproductive apparatus, showing levels of apoptosis before and after *Wolbachia* removal. Apoptosis remains a rare event during germline maturation, and is developmentally programmed during embryogenesis. After *Wolbachia* depletion, cumulative apoptosis is observed in germ cell, embryo and microfilaria. The absence of *Wolbachia* (from the hypodermal chords and from the few embryonic cells derived from the C blastomere) leads to a massive non cell-autonomous, “bystander” apoptosis, in embryonic cells normally devoid of *Wolbachia* (green nuclei).

Regulation of host cell apoptosis is common to many intracellular bacteria [19]–[21]. In many cases the bacterial pathogens commandeer conserved apoptotic regulating cascades. This requires the pathogen to reside within or be directly associated with the affected cell. A unique feature of *Wolbachia’s* anti-apoptotic effect is that there is no correlation between *Wolbachia*-populated cells and cells that undergo apoptosis upon *Wolbachia* depletion (Figure 9). For example, *Wolbachia*-laden hypodermal chords do not undergo apoptosis upon *Wolbachia* depletion. In contrast, while only a few cells in the early embryo (the C linage, [12]) possess *Wolbachia*, the majority of cells in the embryo undergo apoptosis. Similarly, while only the middle third of intrauterine ‘stretched’ microfilariae are *Wolbachia*-infected, apoptosis occurs throughout the entire organism upon *Wolbachia* depletion (Figure 9). These data clearly demonstrate that the effects of *Wolbachia*-depletion on apoptosis are non-cell autonomous.

The non-cell autonomous behaviour implies that *Wolbachia*-infected cells suppress apoptosis in distant non-neighbouring uninfected cells. The source of the *Wolbachia*-infected cells, which are capable of this action-at-distance apoptosis suppression and when in development this occurs remains unclear. Given the abundant *Wolbachia* populations in the female lateral chords, it is likely that these are primarily responsible for apoptotic rates in the oocytes, embryos and intrauterine ‘stretched’ microfilariae. This view is supported by the anatomy of the female in which the lateral chords reside in close proximity to the ovaries and uteri [1], [12] and the greater needs of embryo requirements compared to spermatozoa. Further evidence to support the predominant role of depleted chord cell *Wolbachia* populations in inducing apoptosis in intrauterine developmental stages comes from our observations on released microfilariae. Although microfilariae recovered from tetracycline treated animals did show an increase in the proportion of parasites with medium and high levels of apoptosis, this was not as extensive as we observed with intrauterine ‘stretched’ microfilariae. We were unable, however, to distinguish between microfilariae that were released prior to or after treatment and further experiments using isolated microfilariae only infections are planned to confirm this observation. Our results are, however, consistent with previous observations on the effect of *Wolbachia* depletion on microfilarial viability and transmission. The majority of microfilariae depleted of *Wolbachia* retain their motility and viability in the mammalian host but their ability to develop in the vector is compromised [15].

### Mechanism of apoptosis induction

Our studies raise the issue of the mechanism by which *Wolbachia* suppresses apoptosis. Studies of the influence of other intracellular bacteria on host cell apoptosis demonstrate this may be achieved either by direct targeting of the apoptotic signaling cascade or indirectly by influencing host metabolism [19], [22], [23]. An example of the former occurs in *Chlamydia*, which produces a protein that targets the host pro-apoptotic protein Bim for proteasome-based destruction [24]. *Anaplasma phagocytophilium,* a close relative of *Wolbachia,* inhibits host cell apoptosis through targeting multiple apoptotic pathways [21] and releases a protein, Ats1, via the type IV secretion system that reduces the sensitivity of mitochondria to apoptotic signals [25]. Potential molecular candidates from *Wolbachia,* which have been shown to inhibit apoptosis, include *Wolbachia* surface protein (WSP) [26] or lipoproteins such as wBmPAL, which drive innate and adaptive inflammatory immunity associated with disease pathogenesis [27] and can inhibit apoptosis of human neutrophils [28] (Taylor et al. unpublished observation). Bacterial pathogens subvert apoptosis to enhance their ability to replicate and survive in host cells, whereas, *Wolbachia*, which has a mutualistic rather than a parasitic or pathogenic association with its host nematode, clearly benefits both itself and it’s host by preventing apoptosis during larval, germline and embryonic development to ensure its transmission to the next generation. The only other example of a mutualistic association with *Wolbachia* and arthropods is the parasitic wasp *Asobara tabida* in which depletion of *Wolbachia* results in increased apoptosis in the nurse cells of the female germline [29], suggesting these mutualistic *Wolbachia* associations may share common pathways.

*Wolbachia’s* effect on apoptosis may be through more indirect mechanisms such as influencing the provisioning of essential metabolites, a process likely to be compromised by the hypodermal cytoskeletal defects following *Wolbachia* depletion. In this context it is notable that the nematode biological process most sensitive to *Wolbachia* depletion, embryogenesis and larval development, are processes with high metabolic demands for tissue development, growth and organogenesis. Examples of metabolite provision dependent on *Wolbachia* under nutritional stress have been described in *Drosophila* showing that *Wolbachia* up-regulates bacterioferritin, an iron chelator, and may buffer the host from dramatic changes in iron concentration in the environment [30], [31]. Thus, *Wolbachia* may provide protection from oxidative stress produced by high internal concentrations of iron. Support for a similar protective mechanism operating in filarial nematodes comes from genomic studies demonstrating that *B. malayi* lacks the heme biosynthetic pathway while the *Wolbachia* genome retains most of the entire heme and riboflavin pathways [32]. Heme and riboflavin are efficient iron chelators. Thus the abundance of *Wolbachia* in the chords has a tremendous iron buffering potential.

Together our observations provide a cellular mechanism to explain some of the anti-filarial effects, which occur following antibiotic depletion of *Wolbachia* from filarial nematodes. The pattern of apoptosis we observe closely correlates with the outcomes previously observed in antibiotic therapy in laboratory models of filarial nematodes and in human field trials with doxycycline [5]. The first event, which occurs soon after bacterial depletion from adult female worms, is the blockage of embryogenesis and cessation of microfilarial production, which is consistent with the extensive and profound apoptosis observed in uterine embryonic stages of *B. malayi*. This results in long-term sterilization of adult female worms and the slow decline in microfilarial levels, until patients reach a sustained amicrofilarial state, with benefits to disease reduction in onchocerciasis and interruption of transmission in both onchocerciasis and lymphatic filariasis. This state of sterilization persists for at least 21 months, which is reflected in the retention of widespread apoptotic cells in adult female germline and uterine cells of *O. volvulus* obtained from patients treated with doxycycline, which suggests this is a permanent status. The increase in the proportion of released microfilariae with medium to high levels of apoptotic cells, but less extensive than that observed in intrauterine ‘stretched’ microfilarial stages, is consistent with the observation that following *Wolbachia* depletion, a proportion of microfilariae retain their motility and viability, yet their capacity to develop within the vector host is reduced. A similar pattern of activation of apoptosis in *Wolbachia*-depleted L4 larvae suggests that this process also accounts for the arrested development of L4 larvae to adult worms. Finally, we also observed that apoptosis is not widely induced in the nuclei of somatic tissues and the syncytial chord cells of adult worms, where the majority of the bacterial population exist, which is again consistent with the long-term macrofilaricidal effects seen in both lymphatic filariasis and onchocerciasis, which only manifest after 12 months or 18–27 months respectively. Further studies to define the process by which *Wolbachia* regulates host nematode apoptosis may provide alternative targets to screen for drugs or biomarkers of anti-*Wolbachia* activity, which could yield alternative and improved therapeutic options for the control and elimination of lymphatic filariasis and onchocerciasis.

## Materials and Methods

### Ethics statement

Human parasite material was obtained from patients enrolled in a double-blind placebo-controlled randomized clinical trial conducted in Cameroon. The experimental protocol for this study was designed in accordance with the general ethical principles outlined in the Declaration of Helsinki. The trial was approved by ethics committees of the Tropical Medicine Research Station, Kumba and the Research Ethics Committee of The Liverpool School of Tropical Medicine. Written informed consent was obtained from all participants, with the exception of those who were illiterate, where a literate witness signed on behalf of the participant and the participant added a thumbprint. The trial is registered with the current controlled trials registry, no: ISRCTN48118452.

The animal experiments were carried out in strict accordance with the Animals Scientific (Procedures Act) 1986 (UK) under a license granted by the Home Office (London, UK). Experimental procedures were reviewed and approved by the Animal Welfare Committee, Liverpool School of Tropical Medicine and the Home Office (London, UK).

### Parasite material

Adult *B. malayi* cultivated in the peritoneal cavity of jirds (*Meriones unguiculatus*) for were obtained from TRS Laboratories (Athens Georgia). In Liverpool, infected animals received tetracycline at 2.5 mg/ml in drinking water for a period of 6 weeks. Control infected jirds were maintained in a similar fashion but without the tetracycline. Two weeks after the end of the treatment adult worms and microfilariae were collected from the peritoneal cavities using preheated (37°C) culture medium RPMI-1640 supplemented with 100 U/ml penicillin, 100 mg/ml streptomycin, 2 mM L-glutamine, 2.5 mg/ml amphotericin B, and 25 mM HEPES (GIBCO). A further two groups of jirds were infected with 500 infective third-stage larvae (L3) and L4 larvae were collected 14 days after the jirds were treated with tetracycline (2.5 mg/ml) in drinking water or as untreated controls. All parasites were washed in PBS and either fixed for confocal microscopy or processed for RNA and protein. Ethanol-fixed paraffin-embedded onchocercomas were obtained from patients infected with *O. volvulus* enrolled in a double-blind placebo-controlled randomized clinical trial conducted in Cameroon [13] (trial registration number ISRCTN48118452). Patients received doxycycline for 6 weeks (200 mg/day) or placebo. Nodules were removed surgically after 21 months from the start of the trial.

### Immunofluorescent microscopy

*B. malayi* material was stained for actin and DNA with a fluorescent phalloidin and propidium iodide respectively. An anti-acetylated histone H4 (1∶300, Upstate) was used to stain the host chromatin (*B. malayi* chord nuclei). These fluorescent and immunostaining techniques are described in detail elsewhere [12].

### Tunnel assay

*B. malayi* adult worms were fixed by 4% formaldehyde (Sigma) in PBS and stored until used. The adult worms were cut into several fragments in PBS with 0.1% Triton-X100 (PBST), and transferred to 500 µL eppendorf tubes, with heptane (2/3 V), and 5 µL of NP40. Tubes were vortexed, and rotated for 10 minutes before centrifugation for 1 minute at 4,000 RPM. The supernatant was removed and worm fragments washed in PBST. RNAse treatment was performed with the US biological RNAse at 100 µg/ml overnight at 4°C in PBST. After one wash in PBST, an additional permeabilization was performed, with a DNAse free proteinase K (Roche Applied Sciences) at 20 µg/ml in 10 mM Tris HCl pH7.5, for 30 minutes at 37°C. After a wash in PBST, we followed the manufacturer’s protocol for TUNEL staining at 37°C for 1 hour (*In Situ* Cell Death Detection Kit, Fluorescein, Roche), mounted in Vectashield with propidium iodide and observed 24 hours later with a Leica SP2 confocal microscope.

For *O. volvulus* nodules, tissue sections of 4 µm were cut by microtome, mounted by electrothermal bath at 45°C on Poly-L-lysine. Paraffin sections were deparaffinised by xylene (Fisher Scientific) and rehydrated in a series of ethanol with PBS. Then the material was stained with the TUNEL assay kit (apoTACS In Situ Apoptosis Detection Kit, Trevigen) following manufacturer’s instruction. Nuclei containing fragmented DNA and/or denatured cytoplasm of the cells are stained in blue. Cells that are condensed (pyknotic, apoptotic nuclei or apoptotic bodies) exhibit increased Nuclear Fast Red uptake that results in a darker colour of nuclei (Trevigen). Samples obtained from 6 patients from placebo and 6 patients from doxycycline treated groups were analysed. Stained sections were observed on brightfield (light) Olympus BX 60 microscope.

### Gene expression

Total RNA was extracted from adult females by a Trizol-based method [33]. Purified RNA was treated with 1 U DNase I (Epicentre) at 37°C for 30 min followed by inactivation by EDTA. Approximately 5 µg of treated RNA was used as a template for cDNA synthesis performed by SuperScript III (Invitrogen). Synthesised cDNA was treated by RNase H for 20 min at 37°C and stored at −80°C to be used in quantitative reverse transcription PCR (qRT-PCR) analysis. Specific primers for detection of *ced*-3 gene expression level were designed by PrimerPrimier 4.0 program using cDNA of *ced*-3 *B. malayi* (Bm1 42735) as a template: forward primer 5’- tgtgtgcaaaggagatgctta and reverse 5’- caggcttgcaggaaaaagag. The *gst* gene of *B. malayi* is routinely used for an internal control of qRT-PCR [8]. All amplification and fluorescence quantification were performed by a Bio-Rad Chromo 4 real-time PCR Detector (Bio-Rad). Standard curves were generated using 10-fold serial dilutions of measured PCR products of each gene. All qRT-PCR reactions were performed in total volumes of 20 µl containing 10 µl of SYBR Green I PCR Master Mix (QIAGene), 300 nmol of each gene-specific primer, 30 ng of equilibrated cDNA template, and nuclease free water in the following conditions: 95°C for 15 min followed by 30 cycles of denaturation at 95°C for 15 s, annealing at 60°C (*ced*-3) or 55°C (*gst*), and extension at 72°C for 30 s. The abundance of *ced*-3 gene product in a cDNA sample was estimated from its standard curve and normalized against the *gst* transcript abundance in the same cDNA sample. All comparisons were replicated on two biological samples with three technical replicates for each.

### Western blot

Microfilariae and 14 day old L4 larvae collected from treated and un-treated jirds were washed three times in PBS and then lysed with 50 µl of Tissue Extraction Reagent (Invitrogen). Protein concentration was determined by BCA Protein Assay Kit (Thermo Scientific) using BSA for the standard curve. Equal amounts of protein (40 ng) were mixed with LDS sample buffer (NuPAGE, Invitrogen), boiled for 10 min and chilled on ice for 1 min. Then samples were separated via 4–20% gradient SDS - polyacrylamide gel electrophoresis and transferred to PVDF membranes (Millipore). Western blotting of Cell Death Protein-3 was performed using anti-Caspase-3 monoclonal antibody (NEB), as the primary antibody. The secondary anti-rabbit immunoglobulin G labeled by HRP was applied to the membranes and was developed and detected using SuperSignal chemiluminescence (Thermo Scientific) according to the instructions. Developed signals were exposed on film (Fuji). All protein samples from both treated and control groups were placed on the same gel and then transferred on the same membrane. Three samples per experimental group were used to confirm the same pattern of activated CED-3 protein.
